# Socioeconomic Evaluation of Common Bean (*Phaseolus vulgaris* L.) Cultivation in Providing Sustainable Livelihood to the Mountain Populations of Kashmir Himalayas

**DOI:** 10.3390/plants12010213

**Published:** 2023-01-03

**Authors:** Sidra Nasar, Hamayun Shaheen, Ghulam Murtaza, Tan Tinghong, Muhammad Arfan, Muhammad Idrees

**Affiliations:** 1 Department of Botany, University of Azad Jammu and Kashmi, Muzaffarabad 13100, Pakistan; 2 College of Agriculture and Forestry Engineering and Planning, Tongren University, Tongren 554300, China; 3 Guizhou Key Laboratory of Biodiversity Conservation and Utilization in the Fanjing Mountain Region, Tongren University, Tongren 554300, China; 4 Department of Botany, University of Education Lahore, Vehari Campus, Vehari 61100, Pakistan; 5 College of Life Science, Neijiang Normal University, Neijiang 641000, China

**Keywords:** common beans, food security, livelihood support, Kashmir, subsistence farming, sustainability

## Abstract

*Phaseolus vulgaris* L. is the major pulse cultivated and culturally inculcated in the food habits of the locals in the Himalayan mountainous region of Azad Jammu and Kashmir (AJK), Pakistan. The current study was designed to investigate the role of *P. vulgaris* cultivation in providing livelihood support and to evaluate its production and consumption patterns correlated with the household variables in the state of AJK. The socio-economic data was collected from nine bean cultivated areas in six districts of AJK. The data was acquired by administrating a total of 522 detailed semi structured questionnaires from a diverse array of the respondents following the snowball technique focusing on yield, consumption, revenue generation and livelihood support provided by bean cultivation. The results revealed that common bean cultivation provided significant livelihood support to the local mountainous populations with an average annual income of 50.80 $/family. Subsequently, bean production contributed an average annual per capita income of 6.81 $ in the area, which was attributed to the large family size. Local populations showed an average bean production of 33.93 kg/family, whereas the average annual bean consumption was recorded as 31.99 kg/family in the region. Bean crops were recorded to have an average price of $1.49/kg, with significant variations in the study area correlated with local yield. A data analysis indicated a strong correlation in bean production and consumption patterns. Common bean farmers had a very small farm size, averaging 0.24 ha, where 100% of farmers cultivated common beans as an intercrop with Maize as the primary crop. A Pearson’s test (*p* value < 0.05) revealed significant correlations between land holding and bean production as well as consumption, and bean production with annual per capita income. Small farm size, declining soil fertility, low bean pricing and the unavailability of market mechanisms were identified as the major challenges faced by the common bean farmers. It is recommended to employ an integrated bean farming approach to enhance the economic impact of common bean cultivation in the socioeconomic appraisal of the local populations.

## 1. Introduction

*Phaseolus vulgaris* L., (common bean) is the major legume crop having an 85% share in worldwide bean production [1]. Common beans have an annual global yield of over 27 million tons cultivated on 29 million ha worldwide [2], feeding more than 300 million people linked to agricultural economies across the globe [3]. Common beans cultivation holds a great significance for the rural populations of the Himalayan Mountain region. Bean production is a source of considerable revenue generation at the local level for the small-scale subsistence farmers of the region. *Phaseolus vulgaris* is characterized with a rich nutritional profile, being a rich source of protein, fibre, vitamins, and essential minerals [4]. Common beans evolved in the Mesoamerican regions of Mexico and Guatemala, from where the bean varieties spread in different regions of the world [5]. Common beans are reported to have been introduced in the Himalayan region during the 16th and 17th centuries by European travellers [6].

The state of Azad Jammu and Kashmir (AJK) is mainly a mountainous region in north Pakistan, having a typical subsistence agricultural lifestyle [7]. *P. vulgaris* is culturally inculcated in the food habits of the locals and is an essential dietary commodity consumed with cereals [8]. The cultivation of traditional bean genotypes has been a persistent practice in the Kashmir region as an intercrop with the key cereal crop maize [9]. *P. vulgaris* cultivation contributes significantly to achieve economic sustainability by ensuring the increased income and food security in the region [10].

*P. vulgaris* is a water efficient crop which improves soil health and productivity, making it a climate smart practice. Small land holders in the Himalayan mountainous region still prefer to grow local landraces for their self-consumption as well as revenue generation [11]. AJK is an environmentally sensitive mountain region where prevailing small scale agricultural practices are vulnerable the issues including soil degradation, erosion and climatic extremes [12], whereas the local populations are affected by small land holdings, low income, and poor health, and nutrition [13]. As per sustainable development goals, it is inevitable to attain the sustainable livelihoods by opting for all the available options and exploiting the local potentials in the region [3]. In such scenarios, cultivation of cash crops holds a key importance for the rural mountain farmers due to significant economic benefits [14,15]. Common beans are a right niche cash crop in the Himalayan highlands being used profitably in the consumptive market as an indigenous product, allowing easy access to the local food market [16,17].

The potential of common bean cultivation in the Kashmir Himalayas to contribute towards the sustainable development and socioeconomic appraisal of the rural mountain populations has not been investigated so far, indicating a significant knowledge gap. The current study was designed to investigate the production and consumption patterns of *P. vulgaris* in the state of AJK and evaluate its contribution to the support of the livelihood of the local mountainous population. The specific objectives of the study included (i) the investigation of the common bean production as well as consumption patterns; (ii) to quantify the per capita income generated by the common bean cultivation; (iii) to analyze the farmer’s family and farm size dynamics in correlation with common bean cultivation practices in the rural mountain communities of the state of AJK, Pakistan.

## 2. Results

### 2.1. Common Bean Cultivation System in the Study Area

The farmers in the AJK region were instructed to practice a fixed field agriculture system, which involves crop plantation in all the fields every year following a simple crop system focusing only on Maize cultivation as the primary cereal crop. Common beans are cultivated as an intercrop with maize in summer or the locally called “Kharif season”, which extends from April to October. Bean farming field activities including ploughing, harrowing, levelling, and harvesting were carried out by relying solely upon human and livestock muscle power. The beans are harvested in Late September/Early October in the study area. The seeds are excavated from the pods and cleaned thoroughly. The seeds are then sun dried for 5–10 days depending upon the local climate and temperature until the moisture is completely removed. The dry beans are then put in cloth sacs or household containers for storage for self-consumption or transported to markets.

### 2.2. Gender Based Division of Labour in Common Bean Cultivation

Although both men and women were found to participate significantly in bean cultivation and processing (Table 1), a clear division of labour was recorded among the genders. The tasks involving muscle power and travel including the field preparation, which includes ploughing, harrowing, and weeding along with transport to marketplaces for sale, were mainly carried out by men. The sophisticated and technical tasks of legume harvesting, seed cleaning and drying, storage and cooking were allocated mainly to the females, and requires skill, practice and knowledge of the practices rather than power.

### 2.3. Age Group, Family Size and Literacy Rate

The analysis of age group distribution of the common bean cultivating farmers showed an average age of 51.29 years, ranging from a minimum of 45.5 to a maximum of 55.56 years. A majority of the farmers belonged to the eldest age groups, i.e., 37.3% in the 41–50 years group followed by 32.2% in the 51–60 years group, and 14.3% in the ≥60 years group, whereas there was only 13.8% in the 31–40 year group. Local farmer populations were characterized by a large family size of 7.63 ± 0.42 persons in the whole study area, varying from a minimum of 5.53 to a maximum of 9.58 persons. The highest percentage of farmers (55.2%) had a family size of 6–8 followed by 18.4% with a family size ≤5, 16.1%, with 9–11 persons, whereas 10.3% had ≥12 persons. A majority (66.7%) of the bean cultivating farmers were male, whereas 33.3% were female. A literacy level analysis revealed that 37.4% of the farmers had no formal education, 20.1% had a primary education, 17.8% had a middle-level education, and 10.3% had a secondary-level education. Only 14.3% of farmers had a college-level education. (Table 1).

### 2.4. Farm Size, Farmer’s Experience, and Preference

A socioeconomic survey revealed that a majority (66.1%) of the farmers cultivated common beans as a cash crop for revenue generation. In contrast, 33.9% of farmers cultivated a bean crop for self-consumption. The local agricultural system was found to have a very small farm size, averaging as 0.242 ha per family. A majority of the common bean farmers (43.6%) had very small land holdings, with a farm size of 0.15–0.20 ha followed by 36.8% having 0.05–0.10 ha and 19.5% having a farm size of 0.25–0.30 ha. The average farming experience of the bean farmers was recorded as 19.30 years. A majority of the farmers (45.3%) had farming experience of 11–20 years followed by 32.9% with 21–30 years of experience, 20% having 5–10 years of experience, and 4.11% having experience of >30 years (Table 1).

### 2.5. Common Bean Production and Consumption Pattern

The results revealed an average annual common bean production of 34 ± 6.63 kg per household in the study area. A significant variation in the production was recorded within the study sites, with the highest value of 79.5 ± 11.57 kg recorded at Halmat, whereas a minimum of 11.7 ± 1.52 kg was recorded at Nagder. Annual bean consumption was found to be 32 ± 2.89 kg per household. The highest consumption was recorded at Halmat, at 44.05 ± 2.76 kg, whereas the lowest was at Kel, with 17.4 ± 2.15 kg per family (Table 2). The families with a consumption in excess of their own harvest purchased the beans from the market as per their demand, indicating that the consumption rate was independent of the production. Common beans were recorded as being used both as fresh beans cooked as vegetables along with the legume pod, in addition to being used as dry beans cooked as pulses.

### 2.6. Bean Price, and Annual Family and per Capita Income (US $)

**Figure 1:**
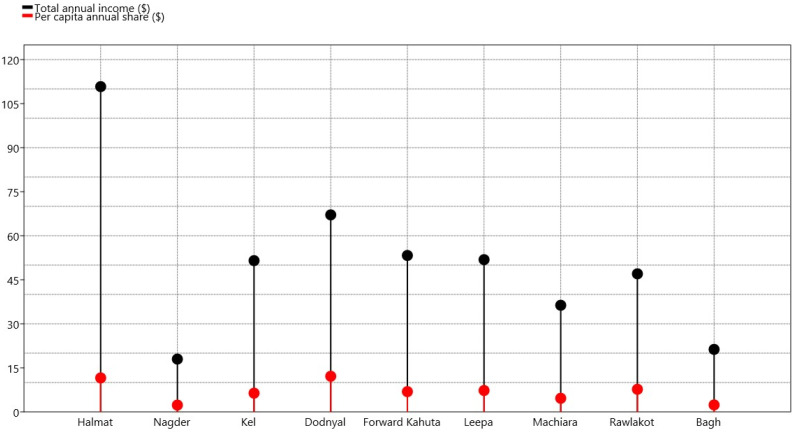
Annual per family as well as per capita income (in US $) generated specifically by common bean cultivation by the farmers in the AJK region.

Results revealed that common bean production provided the families with an average annual income of 50.8 ± 9.16 $/family in the region, i.e., the sum purely earned by the bean cultivation. The highest income was recorded at the Halmat site ($111 ± 13.1/family), whereas the lowest income of $18 ± 4.7/family was recorded at Nagder. However, this per family income showed further variations as family size varied significantly in the study area, thus resulting in a different per capita income pattern as compared to average annual family income. Analysis revealed that common beans provided an annual Per capita income of $6.9 ± 1.16 to the local populations of the AJK region. The highest per capita income was recorded in Dodnyal at $12.14, followed by Halmat ($11.5), whereas the lowest per capita income of $2.32 was recorded for Nagder (Figure 1). The average common bean price was recorded as $1.49 ± 0.02/kg in the study area. The highest bean price of $1.62/kg was recorded at the Dodnyal site, whereas the lowest was at the Halmat site, at $1.40/kg (Table 2).

**Figure 2:**
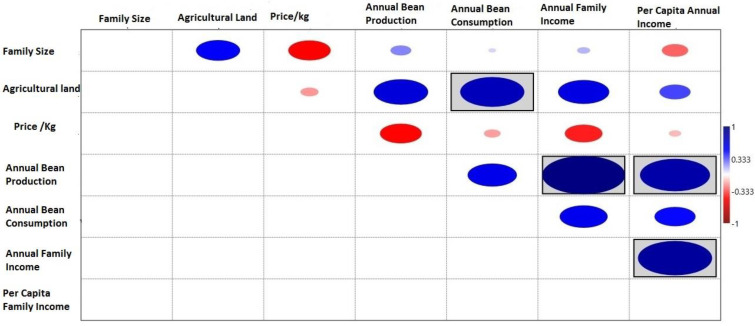
Pearson’s (*p* < 0.05) linear correlation among the common bean production, income and socioeconomic variables.

**Figure 3:**
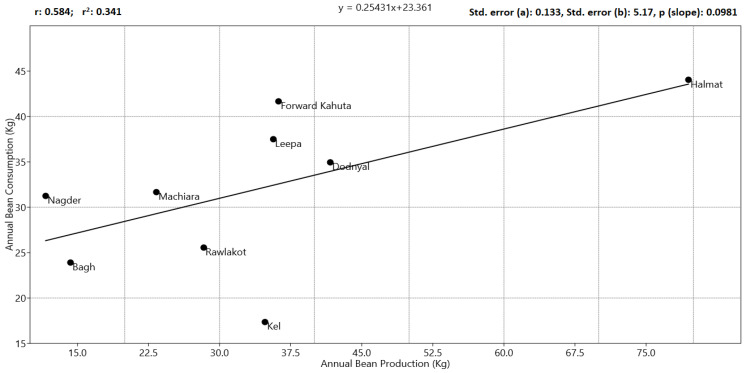
Generalized linear model regression plot of common bean production vs consumption patterns in the AJK region.

A Pearson’s linear correlation test (*p* < 0.05) revealed significant trends among the investigated socioeconomic variables and bean production and consumption patterns in the region. The agricultural land holding showed a significant positive correlation with bean production and consumption pattern, as increased land availability resulted in higher yields. Subsequently, higher bean production also revealed a strong affinity with the annual income of the farmers, both at an individual as well as at the family level. (Figure 2). A linear model shows that the self-consumption of the beans as food in the farmer families exhibited a significant correlation with the bean production pattern (Figure 3).

### 2.7. Major Challenges in Bean Production

**Figure 4:**
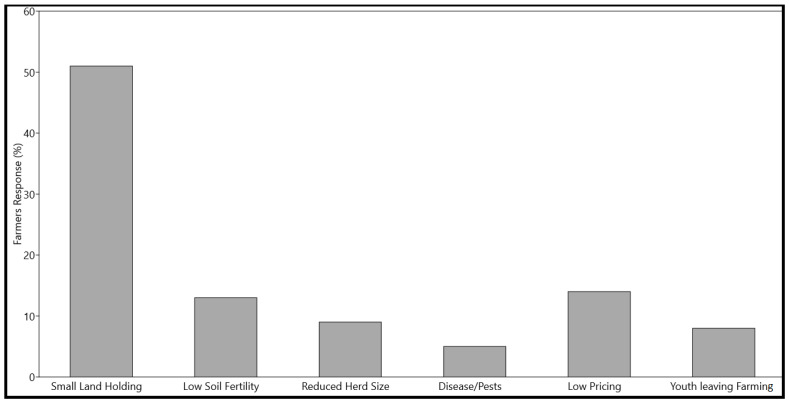
Farmers’ responses regarding the major challenges faced in the bean production in the AJK region.

Fifty-one percent of the farmers considered limited available agricultural land holdings as the biggest challenge for bean cultivation in the region. About 14% of the farmers attributed low bean pricing and the unavailability of transport mechanisms and marketing linkages to connect their production to the major markets of the region as the major challenge. According to the farmers, the isolation of the local markets from the major markets results in low pricing and lesser financial benefits. About 13% of farmers were of the view that low soil fertility is the major reason limiting bean production in the region. Reduced herd size resulting in less natural fertilizers (dung) available for farming was considered as a major issue by 9% of the farmers. Eight percent of farmers believed that the younger generation turning away from the farming practices as a livelihood is a major issue, whereas 5% considered disease and pests as major limiting factors for bean production (Figure 4).

## 3. Discussion

The agricultural system in the mountainous region of the Kashmir Himalayas is based upon very small land holdings, with terraced and fragmented slopes with more than 90% agricultural area being rain fed [18]. Common bean cultivation is a low-cost approach which significantly enhances the farm income of poor mountain farmers in the AJK region. Beans are suitable for inclusion in Maize cropping systems attributed to the socioeconomic and environmental gains leading to an increased crop diversity along with enhancing the soil fertility [19]. This has resulted in an increasing trend of the local subsistence farmers towards bean cultivation for attaining sustainable livelihood and food security [20]. Bean cultivation follows a conservative approach, strictly relying upon the local landraces due to cultural values and better performance in the mountainous climate [21].

Our results revealed an average annual family income of USD $50.8 with a per capita share of USD $6.60 generated specifically by common bean cultivation. A survey in 2016 revealed that 60% of the population in the AJK region have an annual income of just USD $300 per household. This developing region has an annual gross national income just above USD $1000 [22,23]. In this scenario, it is evident that the common bean cultivation offers vital livelihood support to the farmers and contributes significantly to improving their quality of life [24]. This is also evident from the fact that about 66.09% of the interviewed farmers were cultivating common beans as a cash crop. The annual income per family showed broad variations in the study area, ranging from USD $110.8 ($11.57 per capita) to a minimum of $17.99 ($2.32 per capita). These variations are strongly correlated with the available cultivated area, annual yield, and family size, where the farmers with larger farms and small families earn more than those having small land holdings and large families [19,25].

Bean production appeared to be synchronized with the consumption pattern, reflecting a small-scale self-sufficiency in the common bean production at major bean producing areas including Nagder, Taobut and Halmat. This reflects the farmers’ preference for bean cultivation as based upon cultural values [14]. Annual bean production in the region is significantly lower than the neighbouring bean producing countries such as India and China [26], and this is attributed to the very small farm size in the study area. Common bean prices varied at different sites correlated with production and consumption patterns; mainly due to limited productivity and marketing [27]. The local subsistence agriculture is vulnerable to the frequent price fluctuations and low yield that may lead towards market insecurity, ultimately affecting farmers’ crop selection [28].

Farmers in AJK strictly adhere to maize cultivation as the primary crop, as it provides them with grain along with maximum biomass for fodder [29]. However, Maize has much lower cost ($0.3–0.4/Kg) as compared to the common beans price ($1.3–1.6/Kg) due to which it remains insignificant to contribute towards the economic growth of the family unit [30]. In this scenario, bean cultivation as an intercrop with maize considerably enhances revenue generation.

Large family sizes in the study area makes it difficult for the farmers to make ends meet. Large family size may be associated with the polygamous nature of the society, with an interesting implication of increased availability of labour to boost bean production [31,32]. The populations exhibited low literacy rates, which reflects that the farmers lack access to advanced agricultural methods, mainly relying on the conservative farming system [33].

The typical cold Himalayan Mountain climate appeared to have a close connection with the persistence of bean cultivation and consumption in the population of AJK. Geographic analysis revealed that maximum bean production as well as consumption was recorded in the high altitude (>1500 m) sites characterized with cold temperate climates where people culturally prefer common bean usage as a rich energy and protein source [34]. Synchronized with the production, annual bean consumption also followed the same pattern, being maximum in temperate areas (Figure 4). The traditional bean cultivation has interestingly resulted in the maintenance of a diverse range of indigenous varieties in the region, as the repeated use of local seeds in geographically distinct sites avoids germplasm introgression [25,35].

Limited agricultural land availability in the mountainous Kashmir region was identified as a major challenge limiting the production in the region, as the average farm size was very low (<0.2 ha). Farming on terrace fields made on steep mountains limits the use of machinery due to slope and reduced area, forcing framers to rely on muscle power [15]. Bean cultivation is often carried out at marginal lands due to land scarcity, which contributes to poor productivity and results in low yields. Farmers in the region have limited access to the markets and technological services due to remoteness and illiteracy, which prevents them from developing market chains and getting pricing information [36,37]. Socioeconomic issues including changing perceptions of youth leaving the farming practice as well as low literacy and poverty also adds to the severity of the conditions in these Himalayan highlands, which, combined with climatic harshness, becomes a huge challenge for these vulnerable microeconomies relying on bean cultivation for their subsistence [38].

## 4. Materials and Methods

### 4.1. Study Area

Geography: The state of Azad Jammu and Kashmir (AJK) is located in the north of Pakistan, having a total area of 13,297 km² stretched over Latitudes 33°–34° N and Longitude 73°–74° E. Topographically, the area is hilly and mountainous, and is characterized by deep valleys and mountains, and harbors a diversity of agroclimatic zones attributable to the geographic spread and altitudinal gradient from 400 m in the Southern plains adjacent to Punjab to the snowy Himalayan peaks up to 6000 m in the North [39].

Climate: The climatic conditions of the state show huge variations synchronized with the geography, having hot-humid subtropical conditions in the southern low altitude zone, whereas the northern mountainous zone has a cold temperate climate type. Common bean cultivated areas are located in an altitudinal range of 1200–2500 m, having a temperate climate. June and July are the hottest months, with maximum temperatures of approximately 35 °C, whereas December and January are the coldest months, with night temperatures below 0 °C. The average annual precipitation is around 1100 mm, about half of which is received as monsoon rains in July and August [40].

Agricultural System: The state of AJK has a population of 4.45 million, with an annual growth rate of 2.9%, and approximately 91% of the population lives in rural areas. The region has a centuries old culture of subsistence agriculture, with small land holdings and livestock which has a considerable (20–25%) share in the livelihood earnings of the rural populations [41]. Being a mountainous region, only 13% geographical area i.e., 196704 hectares is cultivable land, of which about 92% of the area is rain-fed. Approximately 87% of households have very small land holdings of between one and two acres [42]. Maize and wheat are the major crops cultivated in the region, along with a diversity of vegetables and fruits. Common beans are the only pulse cultivated in the region, and are always grown as an intercrop with maize.

### 4.2. Selection of the Sites and Data Collection

**Figure 5:**
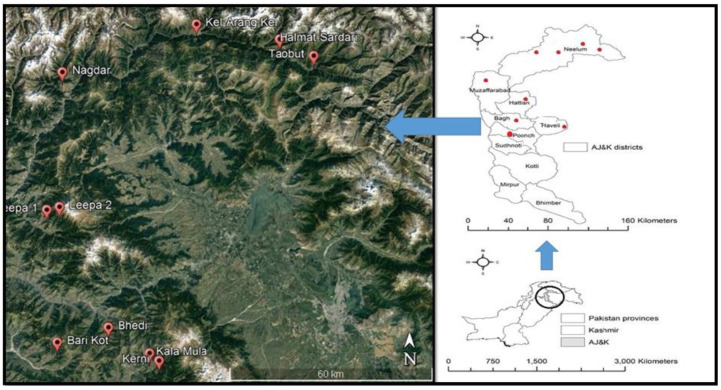
Map of the study area (**right**) and satellite imagery (**left**) of the sampled bean producing locations in AJK.

The socio-economic data about the cultivation practices and role of the common bean in terms of livelihood support and food sustainability was collected from nine bean cultivated areas in six districts of AJK, including Neelum, Muzaffarabad, Hattian, Bagh, Haveli and Poonch (Figure 5).

**Figure 6:**
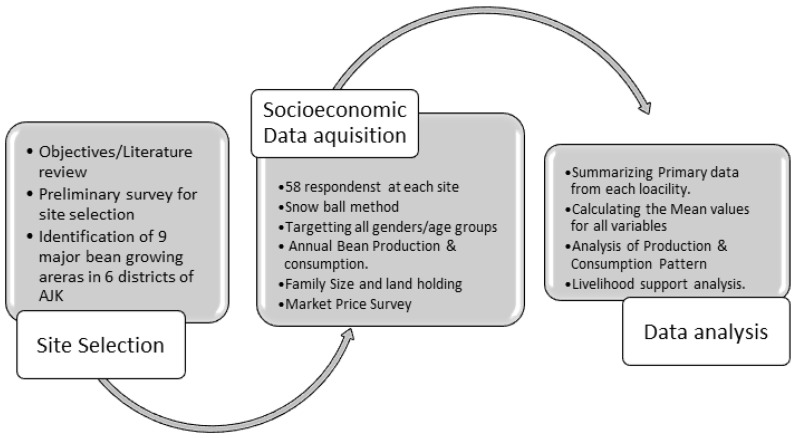
Flow chart of the sampling methodology employed for the collection of socioeconomic data in the bean producing areas of the AJK region.

The bean growing areas were identified after a thorough literature review followed by a preliminary field survey. Four areas, including Halmet, Nagder, Kel and Dodnyal, were selected in the Neelum district, which is the largest bean producing district of the state. The data was acquired by administrating a total of 522 semi structured questionnaires via detailed interviews and discussions from a diverse array of the respondents, involving both genders and different age groups. Interviews were conducted in the local language starting with a brief self-introduction and explaining the objectives of the study to the informants [43]. The snowball method was followed during the primary data acquisition within each site, where every interviewed farmer suggested the next most relevant and experienced farmer to be interviewed [44]. The specific information collected by the survey included informant personal attributes such as age, gender, and education level, followed by the famers’ family attributes including family size, farm size, agricultural preferences, and experience (Figure 6).

The main part of the information focused on common bean cultivation, annual yield, self-consumption, major challenges in bean farming, storage, utilization practices, sale, revenue generation and livelihood support provided by bean cultivation. Common bean price (per Kg) was also recorded from selling households as well as local markets in the area [45].

### 4.3. Data Analysis

The primary data of all the socioeconomic variables, bean production, and consumption was tabulated to calculate the annual as well as the per capita income. The standard error and variance were calculated for the measured values. The data was subjected to multivariate ordination analysis including the Generalized Linear Regression Model and Pearson’s correlation (*p* < 0.05) test to identify correlations among the evaluated socioeconomic variables by using PAST software (Version 4.03) [46].

## 5. Conclusions

A current socioeconomic evaluation revealed that common bean cultivation offers significant livelihood support for the local mountain populations of the AJK region. Revenue generation showed significant variations correlated with farm size, annual yield, and family size. A local scale self-sufficiency in bean production was recorded in the local agricultural practices for self-consumption along with revenue generation as a cash crop. Small land holdings and the absence of market chain and proper bean pricing appeared to be the major challenges faced by the bean cultivating farmers, which should be addressed by following an integrated comprehensive approach in the marginalized farmer communities of the AJK region.

## Figures and Tables

**Table 1:** Socioeconomic variables recorded from the common bean cultivating populations in the study area including age group, family size, literacy rate, land holding and perceptions (respondents: 522).

Sr. No.	Characteristics	Frequency	Percentage (%)
1	Age group		
A	≤30	12	2.30
B	31–40	72	13.79
C	41–50	195	37.36
D	51–60	168	32.18
E	>60	75	14.37
2	Gender		
A	Male	348	66.67
B	Female	174	33.33
3	Literacy level		
A	No formal education	195	37.36
B	Primary	105	20.11
C	Middle	93	17.82
D	Secondary	54	10.34
E	Post-secondary	75	14.37
4	Family size		
A	≤5	96	18.39
B	6–8	288	55.17
C	9–11	84	16.09
D	≥12	54	10.34
5	Years of farming experience		
A	5–10	108	20.69
B	11–20	228	43.68
C	21–30	165	31.61
D	>30	21	4.02
6	Agriculture Land holding (ha)		
A	<0.5	192	36.78
B	0.1–0.2	228	43.68
C	>0.2	102	19.54
7	Farmer’s reason for planting beans		
A	As a cash crop/revenue generation	345	66.09
B	For self-consumption	177	33.91

**Table 2:** Family size, agricultural land, common bean production, consumption and livelihood support recorded from bean producing villages of the AJK region.

Sr. No	Sites	Family Size (Persons)	Agricultural Land (Hectares)	Annual Bean Production (Kg)	Annual Bean Consumption (Kg)	Total Annual Income ($)	Price per Kg ($)	Per Capita Annual Share ($)
**1**	**Halmat**	9.57 ± 0.81	0.44 ± 0.07	79.47 ±11.57	44.05 ± 2.76	110.79 ± 13.10	1.39 ± 0.04	11.57
**2**	**Nagder**	7.75 ± 0.76	0.16 ± 0.03	11.65 ±1.52	31.25 ± 2.38	17.99 ± 4.70	1.54 ± 0.01	2.32
**3**	**Kel**	8.07 ± 0.74	0.15 ± 0.01	34.79 ± 4.61	17.36 ± 2.15	51.52 ± 8.60	1.48 ± 0.05	6.38
**4**	**Dodnyal**	5.52 ± 0.40	0.24 ± 0.04	41.68 ± 4.17	34.95 ± 2.89	67.11 ± 7.51	1.61 ± 0.00	12.14
**5**	**Forward Kahuta**	7.72 ± 0.59	0.36 ± 0.04	36.22 ± 6.08	41.67 ± 2.43	53.28 ± 9.32	1.47 ± 0.03	6.90
**6**	**Leepa**	7.11 ± 0.61	0.21 ± 0.02	35.67 ± 3.85	37.5 ± 2.92	51.86 ± 5.84	1.45 ± 0.03	7.29
**7**	**Machiara**	7.83 ± 0.78	0.31 ± 0.03	23.33 ± 2.46	31.67 ± 2.78	36.31 ± 3.64	1.55 ± 0.02	4.63
**8**	**Rawlakot**	6.11 ± 0.56	0.08 ± 0.01	28.33 ± 2.04	25.56 ± 2.76	47.03 ± 6.04	1.44 ± 0.04	7.70
**9**	**Bagh**	9.00 ± 0.48	0.22 ± 0.03	14.27 ± 1.83	23.91 ± 1.54	21.29 ± 2.97	1.49 ± 0.04	2.37
**10**	**Mean**	7.63 ± 0.42	0.24 ± 0.04	33.93 ± 6.63	31.99 ± 2.89	50.80 ± 9.16	1.49 ± 0.02	6.81 ± 1.16

## Data Availability

The data presented in this study are available on request from the corresponding author.
